# Methane Alleviates Acetaminophen-Induced Liver Injury by Inhibiting Inflammation, Oxidative Stress, Endoplasmic Reticulum Stress, and Apoptosis through the Nrf2/HO-1/NQO1 Signaling Pathway

**DOI:** 10.1155/2019/7067619

**Published:** 2019-11-06

**Authors:** Yang Feng, Ruixia Cui, Zeyu Li, Xia Zhang, Yifan Jia, Xing Zhang, Jinghong Shi, Kai Qu, Chang Liu, Jingyao Zhang

**Affiliations:** ^1^Department of Hepatobiliary Surgery, The First Affiliated Hospital of Xi'an Jiaotong University, Xi'an Shaanxi 710061, China; ^2^Department of Immunology, Shaanxi University of Chinese Medicine, Xianyang Shaanxi 712046, China; ^3^Department of ICU, The First Affiliated Hospital of Xi'an Jiaotong University, Xi'an Shaanxi 710061, China; ^4^Department of SICU, The First Affiliated Hospital of Xi'an Jiaotong University, Xi'an Shaanxi 710061, China

## Abstract

Acetaminophen- (APAP-) induced hepatic injury is an important clinical challenge. Oxidative stress, inflammation, apoptosis, and endoplasmic reticulum stress (ERS) contribute to the pathogenesis. Methane has potential anti-inflammatory, antioxidant, and antiapoptotic properties. This project was aimed at studying the protective effects and relative mechanisms of methane in APAP-induced liver injury. In the *in vivo* experiment, C57BL/6 mice were treated with APAP (400 mg/kg) to induce hepatic injury followed by methane-rich saline (MRS) 10 ml/kg i.p. after 12 and 24 h. We observed that MRS alleviated the histopathological lesions in the liver, decreased serum aminotransferase levels, reduced the levels of inflammatory cytokines, suppressed the nuclear factor-*κ*B expression. Further, we found that MRS relieved oxidative stress by regulating the Nrf2/HO-1/NQO1 signaling pathway and their downstream products after APAP challenge. MRS also regulated proteins associated with ERS-induced apoptosis. In the *in vitro* experiment, the L-02 cell line was treated with APAP (10 mM) to induce hepatic injury. We found that a methane-rich medium decreased the levels of reactive oxygen species (DHE fluorescent staining), inhibited apoptosis (cell flow test), and regulated the Nrf2/HO-1/NQO1 signaling pathway. Our data indicated that MRS prevented APAP-induced hepatic injury via anti-inflammatory, antioxidant, anti-ERS, and antiapoptotic properties involving the Nrf2/HO-1/NQO1 signaling pathway.

## 1. Introduction

Drug-induced liver injury (DILI) is a significantly challenging clinical problem all over the world. According to the recent surveys, DILI is responsible for 25–50% of all acute liver failure cases, and this figure may reach up to more than 50% in America [1]. DILI has the fifth highest mortality rate with an annual incidence of about 19 cases per 100,000 population [2]. Acetaminophen (APAP) overdose contributes to the incidence of more than half of acute liver failure cases and approximately 30% of mortality which is the most common cause of DILI in the western world [3].

The pathogenesis of DILI has not been fully elaborated yet. Various drugs or their toxic metabolites can not only directly affect the hepatic cells, they can also induce excessive inflammation, oxidative stress, and mitochondrial injury, which amplify the damage through apoptosis or necrosis of hepatocytes [4–8]. The toxic metabolite of APAP is N-acetyl-p-benzoquinone imine (NAPQI), which exerts actions on cytochrome P450. NAPQI can be reduced to a nontoxic form by glutathione (GSH) [7]. NAPQI can deplete GSH and covalently bind to the mitochondrial proteins, resulting in the production of free radicals (reactive oxygen species (ROS) and reactive nitrogen species (RNS)). Overproduction of ROS and RNS causes mitochondrial dysfunction, oxidative stress, and cell death [5, 9]. Hepatocyte dysfunction and death cause further stimulation of the other inflammatory cells. The innate immune system is activated and the balance between the pro- and anti-inflammatory cells is jeopardized leading to tissue damage.

Researches have shown that N-acetyl cysteine (NAC), an antidote against APAP poisoning, acts by supplementing GSH and detoxifying NAPQI. NAC is the only proven therapy for APAP-induced liver injury at present. The patients presenting at an early stage of APAP-induced liver injury have better outcomes by NAC than those presenting at an advanced stage [2]. There is a need to explore newer therapeutic options for DILI.

Methane has caught the attention of researchers in recent years due to its unique biological capacities to fight against inflammation, oxidative stress, and apoptosis [10]. Methane is one of the most abundant organic gases present in nature, which has a certain degree of reducibility [11]. Methane is also produced in the human intestine by swallowing air, intestinal chemical reactions, and fermentation of the intestinal methanogens [12]. Administered either by inhaling methane gas or by injecting methane-rich saline, methane has been proven to protect against ischemia and reperfusion-induced organic damage because of its several biological properties [13–18]. Methane can also attenuate carbon tetrachloride- (CCl_4_-) induced liver injury by its anti-inflammatory effects [17]. The role and mechanism of MRS in APAP-induced hepatic injury are still unclear. In this study, the therapeutic effects and relative mechanisms of MRS were studied in APAP-induced hepatic injury to evaluate a new therapeutic option for DILI as well as to broaden the applications of MRS.

## 2. Materials and Methods

### 2.1. Experimental Animals and Cell Line

Male C57BL/6 mice (4–5 weeks old, 21–26 g) were obtained from the Animal Feeding Center of Xi'an Jiaotong University Health Science Center. The mice were kept in an air-conditioned room (22°C, 50% humidity, and 12 h light/dark cycle) and were fed with a standard diet and water *ad libitum*. The experimental procedures were approved by the Animal Care and Use Committee of the Ethics Committee, Xi'an Jiaotong University Health Science Center. This animal work was completed in the Animal Feeding Center of Xi'an Jiaotong University Health Science Center.

The human nontumor hepatic L-02 cell line (Shanghai Institute of Biochemistry and Cell Biology, Chinese Academy of Sciences) was cultured in Dulbecco's modified Eagle medium (DMEM) supplemented with 10% fetal bovine serum (FBS), 100 U/ml penicillin, and 100 U/ml streptomycin in a humidified atmosphere containing 5% CO_2_ at 37°C.

### 2.2. Preparation of Methane-Rich Saline (MRS) and Methane-Rich Medium (MRM)

Methane was dissolved in 0.9% saline and DMEM (with 10% FBS) for 4 h at a 0.4 MPa pressure to produce supersaturated MRS and MRM. The prepared MRS and MRM were stored and sterilized as previously described [19]. MRS and MRM were freshly prepared 1 day before the experiments. The methane levels in the saline and medium were estimated by gas chromatography (Gas Chromatography-9860, Qiyang, Shanghai, China). The concentrations of the MRS and MRM were approximately 1.2–1.5 mmol/l and 1.4–1.8 mmol/l, respectively.

### 2.3. In Vivo Experiments

#### 2.3.1. Mouse Model of DILI and Experimental Design

The mice were randomly allocated into the following groups (*n* = 6 per group): normal saline (NS) group, APAP+NS group, APAP+MRS (5 ml/kg) group, APAP+MRS (10 ml/kg) group, and APAP+MRS (20 ml/kg) group. Intraperitoneal injection of APAP (400 mg/kg) was used to induce liver injury. After APAP challenge, the mice were administered with MRS (5, 10, or 20 ml/kg) or NS (same dose as MRS) 12 h, and 24 h following APAP administration. The mice were sacrificed 24 h after APAP treatment, and the blood samples were collected from the eyeball extraction. The serum samples were separated by centrifugation 4000 rpm for 20 min at 4°C and stored at −20°C. The livers were immediately removed from each animal and kept at −80°C. The fresh liver tissues were divided into two parts, one for tissue protein extraction and liver enzymatic activity assay and the other for histopathological observation.

#### 2.3.2. Histopathological Analysis

The liver samples were fixed with 4% paraformaldehyde for 48 h. Consecutive liver tissue sections 5 *μ*m thick were cut from the paraffin block. Hematoxylin and eosin (H&E) staining of the liver tissues was fulfilled according to the procedure. The histopathological changes were evaluated in a blinded manner by two researchers. The liver histopathological score was calculated as the sum of the individual score grades from 0 (no change), 1 (mild changes), 2 (moderate changes), and 3 (severe changes) for each of the following 6 items, respectively: cytoplasmic color fading, vacuolization, nuclear condensation, nuclear fragmentation, nuclear fading, and erythrocyte stasis. The total score ranged from 0 to 18 [20].

#### 2.3.3. Estimation of Liver Functions

The serum alanine aminotransferase (ALT) assay kit (C009-2, Nanjing Jiancheng Bioengineering Institute, Nanjing, China) and aspartate aminotransferase (AST) assay kit (C010-2, Nanjing Jiancheng Bioengineering Institute, Nanjing, China) were used for detecting the serum ALT and AST levels according to the manufacturer's instructions.

#### 2.3.4. Enzyme-Linked Immunosorbent Assays (ELISA)

In order to evaluate the inflammatory response to APAP, the serum tumor necrosis factor- (TNF-) *α*, interleukin- (IL-) 6, and interleukin- (IL-) 10 levels were assessed with ELISA kits (Lianke, Hangzhou, China) following the manufacturer's instructions.

#### 2.3.5. Estimation of Hepatic Oxidative Stress

The liver tissue homogenate was obtained to detect the malondialdehyde (MDA), superoxide dismutase (SOD), glutathione (GSH), and glutathione peroxidase activity (GSH-px) by using the relative assay kits from Nanjing Jiancheng Bioengineering Institute, Nanjing, China.

#### 2.3.6. Quantitative RT-PCR Analysis

Total RNA was extracted from approximately 20 mg frozen liver tissues using TRIzol (Invitrogen, Carlsbad, CA, USA) according to the manufacturer's protocol. For miRNA detection, 2 *μ*g of total RNA, miRNA-specific stem loop reverse transcriptase (RT) primers, and M-MLV RT (Promega Corp., Madison, WI, USA) were used. For mRNA detection, the isolated RNA was reverse-transcribed into cDNA using a reverse transcription kit (Takara, Dalian, China). Quantitative real-time PCR was performed with an Applied Biosystems 7300HT instrument and Maxima™ SYBR Green/ROX qPCR Master Mix (Fermentas, USA). The mRNA expression was detected in triplicate and standardized with the 18S mRNA expression. The relative levels were calculated using the comparative-Ct Method (*ΔΔ*Ct method). The sequences of primers were as follows: TNF-*α* (forward 5′-GACGTGGAACTGGCAGAAGAG-3′ and reverse 5′-TTGGTGGTTTGTGAGTGTGAG-3′), IL-6 (forward 5′-CCAAGAGGTGAGTGCTTCCC-3′ and reverse 5′-CTGTTGTTCAGACTCTCTCCCT-3′), and IL-10 (forward 5′-GCTCTTACTGACTGGCATGAG-3′ and reverse 5′-CGCAGCTCTAGGAGCATGTG-3′) [21].

#### 2.3.7. Immunohistochemistry

Immunohistochemical staining was performed to detect the inducible nitric oxide synthase (i-NOS), 3-nitrotyrosine (3-NT), Bax, and Bcl-2 expression levels in the liver [18]. The rabbit anti-i-NOS antibody (Abcam, USA, dilution 1 : 500), anti-3-NT antibody (Bioss Biotechnology, China, dilution 1 : 200), anti-Bax antibody (San Ying Biotechnology, China, dilution 1 : 100), and anti-Bcl-2 antibody (Bioss Biotechnology, China, dilution 1 : 200) were incubated overnight at 4°C. A secondary antibody (detection kits with anti-rabbit IgG, SP9001, Zhongshan Jinqiao Biotechnology, Beijing, China) was incubated for 1 h at room temperature. The results were evaluated in a blinded manner by two researchers. Immunohistochemical staining score was assessed by semiquantitative grades from 0 (negative), 1 (mild changes), 2 (moderate changes), and 3 (severe changes), and the extent of staining was graded based on the percentage of positive cells as follows: 0 (negative), 1 (1–25%), 2 (26–50%), 3 (51–75%), and 4 (76–100%). The final staining scores were derived from the product of the intensity and extent scores, ranging from 0 to 12.

#### 2.3.8. Detection of Liver ROS Activation

Dihydroethidium (DHE) fluorescence was used to detect the ROS levels in the liver tissues. The fresh liver tissues were stored in ethanol and dry ice at −80°C. The tissue sections 5 *μ*m thick were obtained, and the liver cryosections were incubated with DHE (10 *μ*M) for 60 min in the dark and washed with PBS. The images were captured using a fluorescence microscope at 200x magnification. DHE oxidized by ROS in the cells showed red emissions under green wavelength excitation (excitation at 490 nm and emission at 610 nm).

#### 2.3.9. TUNEL Staining

Transferase-mediated deoxyuridine triphosphate-biotin nick end labeling (TUNEL) (11684795910, Roche, Switzerland) staining was performed on the paraformaldehyde-fixed and paraffin-embedded liver sections to detect the hepatic cell apoptosis. The sections were observed under a fluorescence microscope at an excitation wavelength of 480 nm and emission wavelength of 530 nm. The fluorescence intensity was quantified using ImageJ2x software.

#### 2.3.10. Western Blot Assay

The relative protein levels in the liver were detected by western blot. Radioimmunoprecipitation assay (RIPA) lysis buffer was used to extract the total proteins and nucleoproteins (14000 rpm, 15 minutes, 4°C).The lysates were separated using sodium dodecyl sulfate-polyacrylamide gel electrophoresis (SDS-PAGE) after the protein concentration was determined. Following that, the proteins were transferred onto polyvinylidene difluoride (PVDF) membranes. The relative blots were blocked with 8% skimmed milk and incubated with antinuclear factor NF-*κ*B p65 antibody (Cell Signaling Technology, USA, dilution 1 : 1000), anti-NF-*κ*B p-p65 antibody (Cell Signaling Technology, USA, dilution 1 : 1000), anti-TNF-*α* antibody (San Ying Biotechnology, China, dilution 1 : 2000), anti-IL-6 antibody (San Ying Biotechnology, China, dilution 1 : 1000), anti-GRP78 antibody (San Ying Biotechnology, China, dilution 1 : 5000), anti-ATF4 antibody (San Ying Biotechnology, China, dilution 1 : 2000), anti-C/EBP-homologous protein (CHOP) antibody (San Ying Biotechnology, China, dilution 1 : 1000), anti-Bcl-2 antibody (Bioss Biotechnology, China, dilution 1 : 300), anti-Bax antibody (San Ying Biotechnology, China, dilution 1 : 5000), anti-caspase 3 antibody (Cell Signaling Technology, USA, dilution 1 : 1000), anti-Nrf2 antibody (Abcam, USA, dilution 1 : 1000), anti-HO-1 antibody (Abcam, USA, dilution 1 : 10000), anti-NQO1 antibody (Abcam, USA, dilution 1 : 10000), anti-*β*-actin antibody (Abcam, USA, dilution 1 : 5000), and anti-histone 3 antibody (San Ying Biotechnology, China, dilution 1 : 5000) overnight at 4°C. The membranes were incubated with secondary antibodies (HRP-conjugated AffiniPure Goat Anti-Rabbit IgG (H+L), San Ying Biotechnology, China, dilution 1 : 5000) for 1 h at room temperature. The protein bands were quantified by the average ratios of integral optic density followed by normalization to the expression of internal control *β*-actin or histone 3, and the results were further normalized to the control. The protein quantification was performed using ImageJ2x software.

### 2.4. In Vitro Experiments

#### 2.4.1. Cell Treatments

The L-02 cells were seeded into 6-well plates and incubated for 24 h. After attachment, the old medium was removed and replaced with a methane-rich medium. To explore the effects of methane on APAP-induced liver injury, the L-02 cells were divided into five groups according to the replaced medium: (1) normal medium (NM) group, (2) MRM group, (3) 10 *μ*M APAP+NM, (4) 10 *μ*M APAP+MRM, and (5) 10 *μ*M APAP+NM+1 mM NAC. NAC was used as a positive control against APAP-induced cellular injury. After incubation for a further 24 h, the cells were harvested for cell viability, intracellular GSH level, apoptosis assay, and immunoblot analyses.

#### 2.4.2. Cell Viability Assay

The L-02 cells were seeded into 96-well plates and incubated. To evaluate the sensitivity of the L-02 cells to APAP-induced liver injury, the cells were exposed to 5 mM, 10 mM, 15 mM, and 20 mM APAP for 12 h, 24 h, and 36 h, respectively. Further, the cells were incubated with CCK8 for 2 h using a kit (Qi Hai Biotechnology, China). The optical density was measured at 450 nm. The cell viability was calculated as the percentage of control.

#### 2.4.3. Estimation of Intracellular GSH

The cells were lysed in 1 ml of ice-cold PBS containing 0.25% EDTA and centrifuged at 1500 rpm/min for 5 min at 4°C. The supernatants were collected and stored and further used for the determination of intracellular GSH content using a GSH quantification kit (Jiancheng, Nanjing, China) according to the manufacturer's instructions.

#### 2.4.4. Flow Cytometry Assay

The apoptotic cells were detected by Annexin 7AAD/PE apoptosis detection kit (BD Biosciences Inc., USA) following the manufacturer's instructions. The collected cells were washed twice with ice-cold PBS and centrifuged at 1500 rpm for 5 min at 4°C. The cells were analyzed by flow cytometry (BD Biosciences Inc., USA). The apoptotic cell percentage was defined as the sum of the early and late apoptotic cell percentage.

#### 2.4.5. Estimation of Intracellular ROS

The L-02 cells were seeded into 24-well plates and incubated. After attachment, the L-02 cells were incubated with a probe (DHE, 10 *μ*M) for 60 min in the dark and washed with PBS. The images were captured using a fluorescence microscope at 200x magnification. The DHE oxidized by ROS in the cells showed red emissions under green wavelength excitation (excitation at 490 nm and emission at 610 nm).

#### 2.4.6. Western Blot Assay

The protein expression levels in the L-02 cell were detected by western blotting. The detailed procedures have been described previously (Section 2.3.10).

### 2.5. Statistical Analysis

The data were represented as the mean ± standard deviation (SD). All statistical analyses were performed by SPSS version 18.0 (SPSS Inc., Chicago, USA). For comparisons among multiple groups, one-way analysis of variance followed by the Student-Newman-Keuls post hoc test was performed. GraphPad Prism (GraphPad Software, USA) was used to obtain the figures. All the tests were two-sided and a *p* value of <0.05 was considered as statistically significant.

## 3. Results

### 3.1. MRS Alleviated APAP-Induced Liver Injury (Histopathological Changes and Liver Functions)

**Figure 1:**
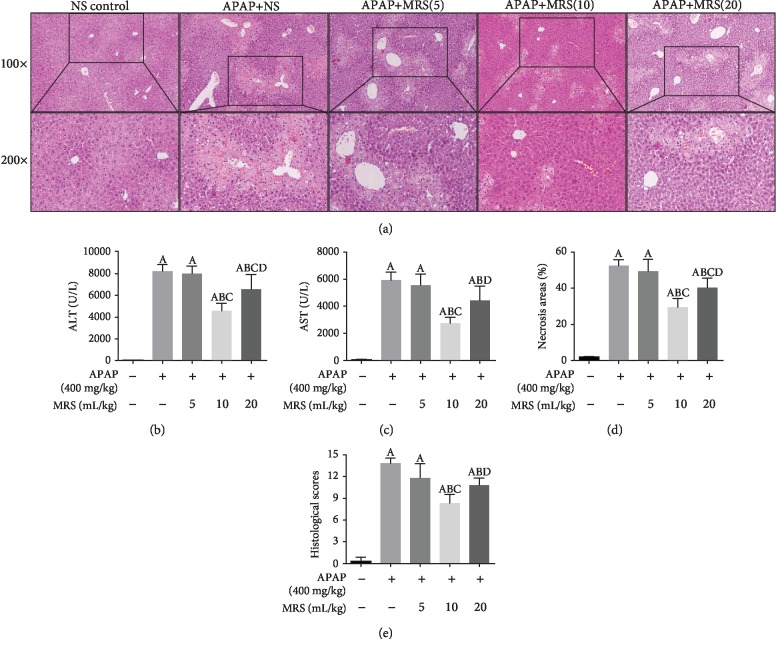
MRS alleviated the APAP-induced liver injury: (a) liver tissues (H&E), (b) ALT levels, (c) AST levels, (d) necrotic areas, and (e) histopathological scores (^A^*p* < 0.05 as compared to the NS group, ^B^*p* < 0.05 as compared to the APAP group, ^C^*p* < 0.05 as compared to the 5 ml/kg MRS group, and ^D^*p* < 0.05 as compared to the 10 ml/kg MRS group).

The effects of MRS on APAP-induced hepatic injury and its optimal effective concentration were evaluated. In the experimental mice, 400 mg/kg APAP successfully induced hepatotoxicity. Pathological changes, such as central necrosis, lymphocytic infiltration, intrahepatic hemorrhagic, and destruction of the liver structure, were observed in the APAP group, which were significantly alleviated in the APAP + 10 ml/kg MRS and APAP + 20 ml/kg MRS groups (Figure 1(a)). A significant reduction was observed in the histopathological score in the APAP + 10 ml/kg MRS and APAP + 20 ml/kg MRS groups in comparison to that of the APAP group (*p* < 0.05) (Figure 1(e)). The results corroborated with the percentage of necrotic areas (*p* < 0.05) (Figure 1(d)). The levels of serum ALT and AST in the APAP + 10 ml/kg MRS and APAP + 20 ml/kg MRS groups were significantly lower than those in the APAP group (*p* < 0.05) (Figures 1(b) and 1(c)). The protective effects of 20 ml/kg MRS, however, was less compared to those of 10 ml/kg MRS as signified by the histopathological scores, necrotic areas, and levels of ALT and AST (*p* < 0.05); 5 ml/kg MRS did not show any significant improvement against APAP-induced liver injury. Considering the optimal efficacy and drug amount, a dose of 10 ml/kg MRS was selected for the successive animal experiments to explore the protective effects and underlying mechanisms of action in DILI.

### 3.2. MRS Reduced the Expression of Inflammatory Mediators Regulated by NF-*κ*B Signaling after APAP Challenge

**Figure 2:**
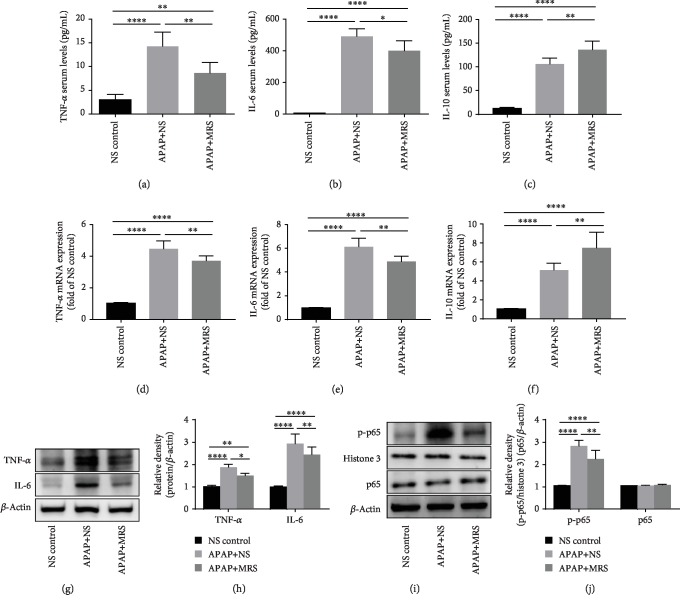
MRS reduced the expression of inflammatory mediators regulated by NF-*κ*B signaling after APAP challenge. (a–c) Serum levels of TNF-*α*, IL-6, and IL-10; (d–f) mRNA expression levels of TNF-*α*, IL-6, and IL-10; (g, h) western blot results of TNF-*α* and IL-6 expression; (i, j) western blot analysis showing p65 and p-p65 expression in the liver (^∗^*p* < 0.05, ^∗∗^*p* < 0.01, ^∗∗∗^*p* < 0.001, and ^∗∗∗∗^*p* < 0.0001).

Inflammation is one of the important causes of progression of DILI [22]. The common cytokines that induce inflammation, such as TNF-*α* and IL-6, were found at significantly lower levels in the MRS group than that in the APAP group (*p* < 0.01 and *p* < 0.05, respectively) (Figures 2(a) and 2(b)). Regarding the anti-inflammatory cytokine factors, ELISA analysis indicated that MRS significantly increased the IL-10 level (*p* < 0.01) (Figure 2(c)). The RT-PCR results of TNF-*α*, IL-6, and IL-10 were consistent with the ELISA assay (*p* < 0.01) (Figures 2(d), 2(e), and 2(f)). Likewise, the protein bands showed that TNF-*α* and IL-6 expression was significantly reduced after MRS treatment (*p* < 0.05 and *p* < 0.01, respectively) (Figures 2(g) and 2(h)). The NF-*κ*B-mediated pathway, a classical pathway of inflammation, was further explored to explain the anti-inflammatory activities of MRS. The relative band intensities of NF-*κ*B p-p65 in the MRS group were significantly lower than that in the APAP group (*p* < 0.01) (Figures 2(i) and 2(j)). These results showed that MRS protected against APAP-induced liver inflammation by inhibiting the NF-*κ*B-mediated pathway.

### 3.3. MRS Relieved Oxidative Stress after APAP Challenge

**Figure 3:**
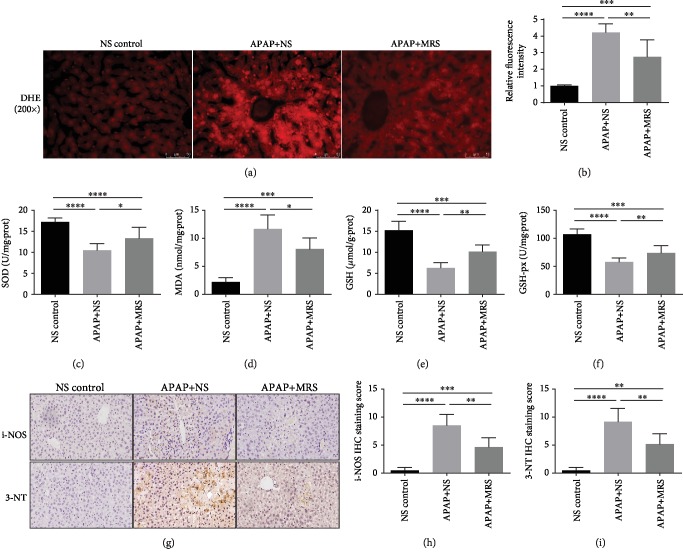
MRS relieved oxidative stress after APAP challenge. (a) Immunofluorescence of DHE expression; (b) relative fluorescence intensity in the liver; (c–f) levels of SOD, MDA, GSH, and GSH-px in the liver; (g) IHC staining (200x) of i-NOS and 3-NT expression in the liver; and (h, i) relative IHC staining scores (^∗^*p* < 0.05, ^∗∗^*p* < 0.01, ^∗∗∗^*p* < 0.001, and ^∗∗∗∗^*p* < 0.0001).

Oxidative stress is a crucial factor in the APAP-induced liver injury. ROS accumulation is the main cause of oxidative stress [23]. The status of oxidative stress caused by APAP was evaluated by measuring the levels of ROS accumulation, antioxidant factors (SOD, GSH, and GSH-px), and oxidative stress factors (MDA, 3-NT, and i-NOS). The DHE fluorescence probe results showed that the ROS fluorescence intensity in the APAP group was significantly higher than that in the NS group (*p* < 0.0001), whereas it was significantly reduced by MRS treatment (*p* < 0.01) (Figures 3(a) and 3(b)). The levels of serum SOD, GSH, and GSH-px were significantly lower in the APAP group than in the NS group (*p* < 0.0001). These levels were increased in the MRS group unlike in the APAP group (*p* < 0.05 or *p* < 0.01) (Figures 3(c), 3(e), and 3(f)). Regarding the oxidative stress factors, MDA analysis indicated that MRS decreased the level of MDA significantly (*p* < 0.05) (Figure 3(d)). Furthermore, immunohistochemistry showed that the relative fluorescence intensities of 3-NT and i-NOS in the MRS group were significantly lower than those in the APAP group (*p* < 0.01) (Figure 3(g)–3(i)). The results above verified the suppressed role of MRS in APAP-caused oxidative stress in the liver.

### 3.4. MRS Inhibited Endoplasmic Reticulum Stress- (ERS-) Induced Apoptosis after APAP Challenge

**Figure 4:**
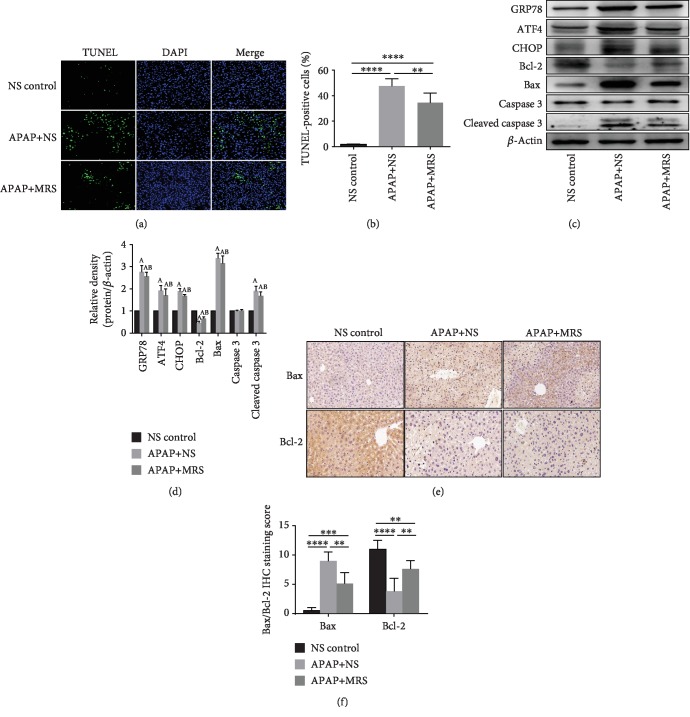
MRS inhibited ERS-induced apoptosis after APAP challenge. (a) TUNEL fluorescence staining (green), nuclear counterstaining (blue), and merging of both channels in the representative liver sections (200x); (b) percentage of TUNEL-positive cells; (c, d) western blot results of GRP78, ATF4, CHOP, Bcl-2, and Bax expressions in the liver; (e) IHC staining (200x) showing Bax and Bcl-2 expressions in the liver; and (f) relative IHC staining scores (^∗^*p* < 0.05, ^∗∗^*p* < 0.01, ^∗∗∗^*p* < 0.001, and ^∗∗∗∗^*p* < 0.0001).

Previous studies have demonstrated that APAP-induced hepatotoxicity is closely related with apoptosis [24]. TUNEL staining of the liver showed a higher number of apoptotic cells in the APAP group, whereas fewer ones were found in the MRS group (Figure 4(a)). Semiquantitative results suggested that there was a significant decrease in the number of apoptotic cells after the APAP-challenged animals were treated with MRS (*p* < 0.01) (Figure 4(b)).

Since ERS stimulates CHOP overexpression and induces apoptosis [25], we hypothesized that ERS- and CHOP-mediated pathways were the potential mechanisms during apoptosis suppression. The expression levels of the ERS biomarkers GRP78 and ATF-4, as well as the levels of apoptosis-related molecules, such as CHOP, Bax, and cleaved caspase 3, were increased in the APAP group compared to the levels in the NS group (*p* < 0.05) (Figures 4(c) and 4(d)). However, MRS reduced the expression of the ERS biomarkers (*p* < 0.05). Bcl-2, an inhibitor of the proapoptosis pathway showed a lower expression in western blot and IHC after treatment with APAP (*p* < 0.05 and *p* < 0.01, respectively), although its expression was upregulated by MRS (*p* < 0.01) (Figures 4(e) and 4(f)). Thus, MRS protected the hepatocytes against APAP-induced liver injury by suppressing ERS-induced apoptosis.

### 3.5. MRS Activated Nrf2-Dependent Protective Antioxidant Mechanisms against APAP Challenge

**Figure 5:**
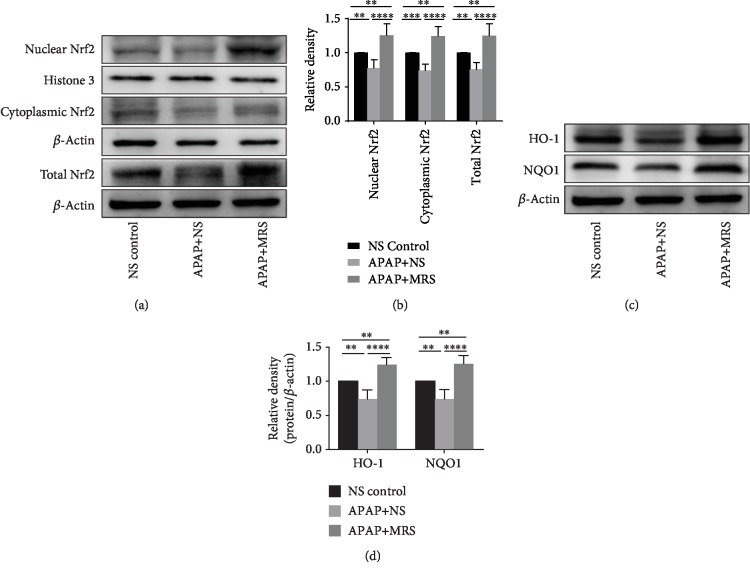
MRS activated Nrf2-dependent antioxidant protective mechanisms after APAP challenge. (a, b) Western blot analysis showing nuclear Nrf2, cytoplasmic Nrf2, and total Nrf2 expression in the liver and (c, d) western blot analysis showing HO-1 and NQO1 expression in the liver (^∗^*p* < 0.05, ^∗∗^*p* < 0.01, ^∗∗∗^*p* < 0.001, and ^∗∗∗∗^*p* < 0.0001).

To explore the possible antioxidant mechanisms of MRS against stress, we assessed the Nrf2 signaling pathway, an important antioxidant response element signaling pathway [26]. As shown in Figures 5(a) and 5(b), Nrf2 expression was decreased in the cytoplasm, nucleus, and the whole cells in the animals treated with APAP (*p* < 0.01). Nrf2 expression was, however, significantly increased with MRS treatment compared to that with APAP (*p* < 0.0001). HO-1 and NQO1 are the downstream molecules of the Nrf2 signaling pathway and were found to have a higher expression by western blot analysis in the MRS group than in the APAP group (*p* < 0.0001) (Figures 5(c) and 5(d)). This indicated that MRS might protect against APAP-induced oxidative stress by activating the Nrf2-dependent pathway.

### 3.6. MRS Protected the L-02 Cells against APAP-Induced Liver Injury by Preventing Oxidative Stress and Apoptosis

**Figure 6:**
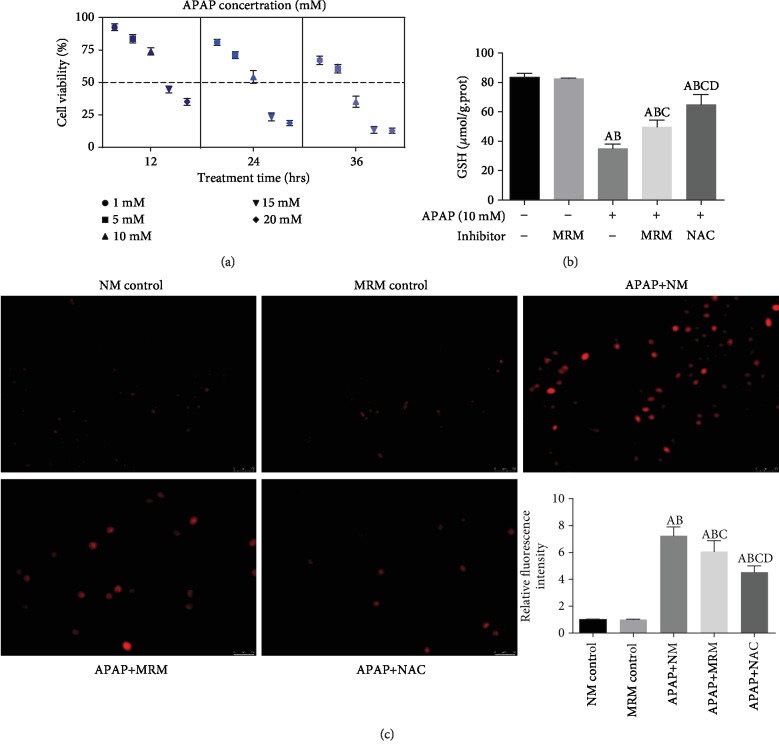
MRS inhibited APAP-induced cellular oxidative stress. (a) Cell viability after treatment with APAP at doses of 5 mM, 10 mM, 15 mM, and 20 mM for 12 h, 24 h, and 36 h exposure, respectively; (b) GSH level in L-02 cells after treatment with 10 mM APAP concentration for 24 h; (c) immunofluorescence of ROS expression and (b) relative fluorescence intensity in the L-02 cells (^A^*p* < 0.05 as compared to the NM group, ^B^*p* < 0.05 as compared to the MRM group, ^C^*p* < 0.05 as compared to the APAP+NM group, and ^D^*p* < 0.05 as compared to the APAP+MRM group).

The L-02 cells were cultured in the study to evaluate the sensitivity of the cells to APAP-induced liver injury in a dose- and time-dependent manner. The cells were exposed to 1 mM, 5 mM, 10 mM, 15 mM, and 20 mM APAP for 12 h, 24 h, and 36 h, respectively. As shown in Figure 6(a), when the cells were treated with 10 mM APAP and exposed for 24 h, the median lethal concentration was obtained, which is a classical marker to estimate the potency of a toxin. Therefore, we used 10 mM APAP for 24 h of exposure to develop an APAP-induced cytotoxic model in the *in vitro* experiments.

**Figure 7:**
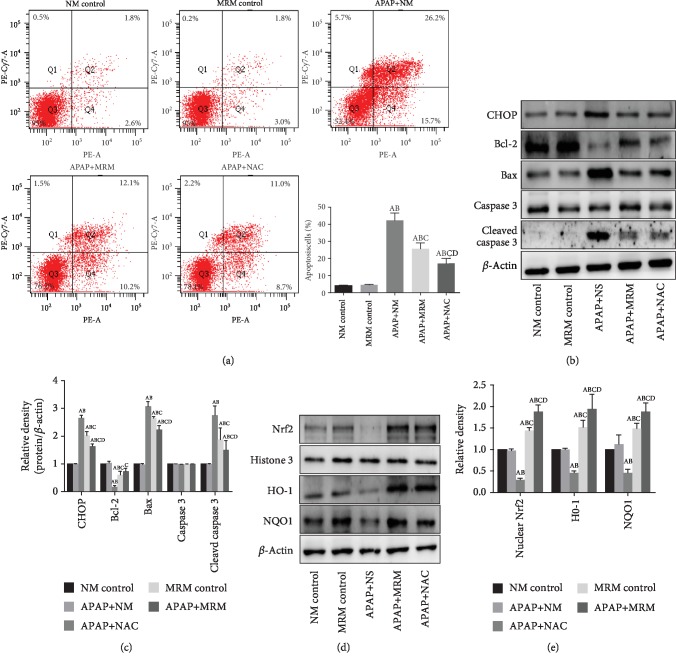
MRS prevented L-02 cells from APAP-induced injury via restraining oxidative stress and apoptosis. (a) Flow cytometry results of the apoptotic L-02 cells; (b, c) western blot results of CHOP, Bcl-2, and Bax expression in L-02 cells; and (d, e) western blot results of Nrf2, HO-1, and NQO1 expression in L-02 cells (^A^*p* < 0.05 as compared to the NM group, ^B^*p* < 0.05 as compared to the MRM group, ^C^*p* < 0.05 as compared to the APAP+NM group, and ^D^*p* < 0.05 as compared to the APAP+MRM group).

The intracellular ROS accumulation and GSH level were estimated for the evaluation of oxidative stress.  Figure 6(b) showed that both methane and NAC had incremental effects on the intracellular GSH level which was suppressed by APAP (*p* < 0.05). Immunofluorescence showed that the intracellular ROS accumulation was reduced significantly when the injured L-02 cells were treated with MRS or NAC (*p* < 0.05) (Figure 6(c)). To further clarify the mechanism, the Nrf2-mediated pathway was evaluated in the L-02 cells. MRS and NAC stimulated the pathway by significantly upregulating the expression of nuclear Nrf2, HO-1, and NQO1 (*p* < 0.05) (Figures 7(d) and 7(e)).

Furthermore, L-02 cell flow cytometry demonstrated that the proportion of apoptotic cells was significantly decreased in both APAP+MRM and APAP+NAC groups compared to the APAP group (*p* < 0.05) (Figure 7(a)). The CHOP-mediated mechanisms were explored *in vitro*. As shown in Figures 7(b) and 7(c), the relative band intensities of the apoptotic proteins CHOP, Bax, and cleaved caspase 3 were significantly increased by APAP (*p* < 0.05), whereas the same were significantly decreased by APAP+MRM and APAP+NAC (*p* < 0.05). The expression levels of Bcl-2, however, showed a reverse phenomenon in the different groups (*p* < 0.05).

In short, MRS mitigates APAP-induced oxidative stress and apoptosis by activating the Nrf2-mediated pathway and inhibiting the CHOP signaling pathway as demonstrated in the *in vitro* and *in vivo* experiments.

## 4. Discussion

DILI is the leading cause of acute liver injury and has been a considerable clinical concern. APAP is a major contributor to DILI, especially in western countries [27]. Methane is the most abundant organic gas found in the earth. Researchers have revealed the therapeutic potentials of methane in concanavalin A- and CCl_4_-induced liver injuries [15, 17]. Here, we evaluated the protective effects and potential mechanisms of action of MRS for the first time in APAP-induced hepatic injury.

APAP caused necrosis and inflammatory infiltrations in the mice liver, along with an elevation of serum ALT and AST levels indicating liver dysfunction [28]. In this study, treatment with MRS alleviated the histopathological lesions of the liver, lowered serum ALT and AST levels, and increased cell viability. We chose 10 ml/kg MRS instead of 20 ml/kg MRS because of the overload of cell edema induced by 20 ml/kg MRS, which was also confirmed by our previous research [29].

Oxidative stress is one of the pivotal mechanisms involved in APAP-induced hepatic injury and is considered as a potential therapeutic target [27]. NAPQI induces mitochondrial oxidative stress and generates ROS. An overproduction of ROS subsequently increases the oxidative stress and leads to a disequilibrium between oxidative stress and level of antioxidants. ROS, lipid peroxidation, and mitochondrial dysfunction lead to nuclear DNA fragmentation in succession and the cells eventually die [30, 31]. In this study, we found that the levels of antioxidants (SOD, GSH, and GSH-px) were increased while oxidative stress factors (MDA, 3-NT, and i-NOS) were lowered by MRS, indicating its antioxidant property. Thereafter, the Nrf2-mediated pathway was analyzed to further explore the mechanism involved in the antioxidant effects of MRS. Previous studies have suggested that Nrf2, an antioxidant transcriptional factor, is involved in the redox homeostasis and plays an essential role in DILI [32]. After evaluating the expression of Nrf2 and its downstream molecules HO-1 and NQO1, we observed that the Nrf2-mediated pathway is suppressed in DILI. MRS showed a protective effect by upregulating the expression of these molecules. The results of the *in vitro* and *in vivo* experiments were in synchrony. This implies that MRS exerted antioxidant effects in DILI by activating the Nrf2-mediated pathway.

Inflammation is another essential factor in the pathogenesis of DILI [7]. The metabolite of APAP stimulates inflammatory cell infiltration and proinflammatory cytokine release, ultimately leading to liver damage [33]. Furthermore, ROS and cell debris are also the crucial triggers of inflammatory mediators in DILI [34]. In our experiment, a higher count of the inflammatory cells and elevated serum levels of proinflammatory cytokines, such as TNF-*α* and IL-6 after APAP administration, were observed, which demonstrated that MRS caused significant inhibition to inflammation. Consistent with the results of Ju et al., IL-10 has protective effects on liver injury. Compared with the APAP group, the MRS may promote more IL-10 secretion [35]. NF-*κ*B is a major transcriptional regulator that provokes the transcription of target genes associated with inflammation response, including the production of inflammatory cytokines [36]. Our results showed that MRS inhibited the APAP-induced activation of NF-*κ*B signifying that the potential anti-inflammatory effects of MRS in DILI were mediated by this mechanism.

In addition, less TUNEL positivity was found after MRS treatment, which indicated that MRS could protect the hepatocytes from APAP-induced apoptosis [5]. Because of the highlighted importance of ERS in cell apoptosis, particularly in the secretory cells rich in ER like hepatocytes [37], the biomarkers of ERS (GRP78 and ATF-4) were studied. MRS downregulated the enhanced expression of GRP78 and ATF-4 in DILI. The activation of ERS is closely related to the overexpression of CHOP, which is the key leading gene causing hepatocyte apoptosis [24, 38]. The present study showed that MRS induced Bcl-2 activation and inhibited the expressions of CHOP, Bax, and cleaved caspase 3. The results with the L-02 cells were also in synchrony. Thus, MRS inhibited apoptosis by suppressing the ERS-mediated pathway.

**Figure 8:**
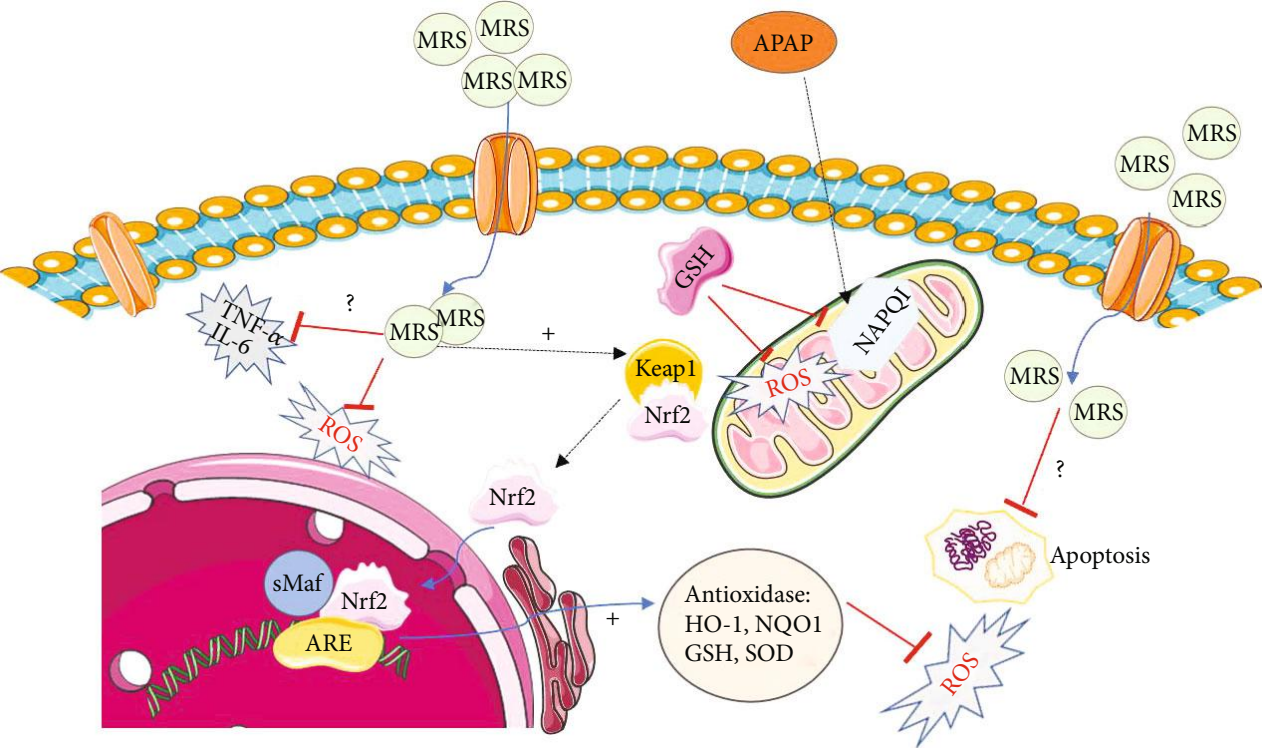
Possible mechanisms of the effects of methane against APAP-induced hepatic injury. APAP induced the production of cytotoxic NAPQI, which induced oxidative stress, inflammation, and cell apoptosis. MRS could penetrate the cell membrane and protect against APAP-induced cell damage by enhancing the Nrf2-mediated antioxidant activity, downregulating NF-*κ*B-mediated inflammation, and suppressing ERS-mediated apoptosis.

MRS exhibited protective effects against APAP-induced hepatic injury by its antioxidant, anti-inflammatory, and antiapoptotic properties. These properties were also proven to be protective against ischemia-reperfusion injury, endotoxin shock, diabetic retinopathy, acute pancreatitis, etc. [10]. In this study, we have hypothesized the possible molecular mechanisms of MRS actions (Figure 8). It remains unclear, however, how methane specifically acts on the hepatocytes. Boros et al. offered a conjecture that the accumulated methane on the cell membrane might impact the physicochemical properties or function of proteins around it influencing the activity of membrane-bound enzymes, especially those resulting in ROS production [13]. Sentiment was also buoyed that methane could exert its effects by modulating the membrane channels similar to the effects of halothane on G-proteins [39]. Chen et al. hypothesized that methane could penetrate the cell membranes and reach the organelles like mitochondria which are responsible for the intracellular production of ROS [14]. This could be explained by the antioxidant effects exhibited by methane. Further studies are required to clarify the mechanism of biological actions of methane.

N-acetylcysteine (NAC) is a well-known antidote for acetaminophen toxicity in the clinic. Cai et al. demonstrated that N-acetylcysteine protects against liver injury induced by carbon tetrachloride via activation of the Nrf2/HO-1 pathway [40]. Hendrickson showed the most appropriate dose of N-acetylcysteine after a massive acetaminophen overdose [41]. Treatment with NAC is limited to a narrow therapeutic window, and other specific therapies against APAP-induced liver injury are urgently needed. In this study, the protective effects of MRS were evaluated in a classic animal model of APAP challenge which may be an ideal complementary therapy for NAC. It is noteworthy to mention that although methane is a nontoxic gas, it is highly inflammable and might explode when comes in contact with air [14]. By contrast, MRS is a comparatively stable formulation of methane, which is safe and convenient to use [25]. In summary, our results revealed that MRS could protect against APAP-induced DILI by activating Nrf2-mediated antioxidant responses, reducing NF-*κ*B-dependent inflammatory responses, and by inhibiting ERS-caused apoptosis, which brings a promising prospect that MRS would be an alternative method for the treatment of APAP-induced DILI.

## Data Availability

The data related to mouse data, cytokine serum and mRNA levels, oxidative stress indictors, TUNEL staining, and western blot images used to support the findings of this study are available from the corresponding authors upon request.
